# Seminal plasma metabolomics approach for the diagnosis of unexplained male infertility

**DOI:** 10.1371/journal.pone.0181115

**Published:** 2017-08-10

**Authors:** Shanlei Qiao, Wei Wu, Minjian Chen, Qiuqin Tang, Yankai Xia, Wei Jia, Xinru Wang

**Affiliations:** 1 State Key Laboratory of Reproductive Medicine, Institute of Applied Toxicology, School of Public Health, Nanjing Medical University, Nanjing, China; 2 Key Laboratory of Modern Toxicology of Ministry of Education, School of Public Health, Nanjing Medical University, Nanjing, China; 3 State Key Laboratory of Reproductive Medicine, Department of Obstetrics, Nanjing Maternity and Child Health Hospital, Obstetrics and Gynecology Hospital Affiliated to Nanjing Medical University, Nanjing, China; 4 University of Hawaii Cancer Center, Honolulu, Hawaii, United States of America; Universite Blaise Pascal, FRANCE

## Abstract

We used a gas chromatography-mass spectrometry (GC-MS) based metabolomics approach to obtain the metabolic profiling of unexplained male infertility (UMI), and identified seminal plasma biomarkers associated with UMI by a two-stage population study. A robust OPLS-DA model based on these identified metabolites was able to distinguish 82% of the UMI patients from health controls with a specificity of 92%. In this model, 44 metabolites were found differentially expressed in UMI subjects compared with health controls. By pathway enrichment analysis, we identified several major changed metabolic pathways related to UMI. Our findings provide new perspective for the diagnosis of UMI.

## Introduction

Declines in fertility have become a serious concern in recent years, and have been accompanied by increasing requirements for infertility treatment [1]. Infertility is clinically defined as failure of a couple to conceive after one year of regular sexual intercourse. An estimated 4–17% of couples seek medical treatment in order to rectify their infertility. It is reasonable to assume that there are many more cases of infertility that are unreported [2]. About 50% infertile cases are attributed to male factor. Despite considerable efforts to identify the pathophysiology of the disease, the underlying mechanisms of approximately half of infertility cases are still unclear.

Unexplained male infertility (UMI) is a diagnosis reserved for men in whom routine semen analysis results are within normal values, and physical as well as endocrine abnormalities were ruled out. Currently, routine semen analysis remains the backbone of the evaluation of the UMI [3]. Technological developments in the field of metabolomics may bring robust and cost-effective clinical tools to fix the shortcomings of the routine semen analysis and provide new insights into UMI. Seminal plasma has long been used as a key source to investigate male infertility. It has been reported that infertile patients represent significant change at metabolome level compared to fertile men, based on metabolomics fingerprinting studying infertile patient seminal plasma [4]. Previous studies have shown that metabolic fingerprinting can be applied as a screening tool for male infertility problems [5].

Further tests are certainly required beyond routine semen analysis for evaluating subfertile men. Genomic [6–9] and proteomic [10–12] efforts were paid in diagnostic and mechanism study for male infertility. As metabolomics is a newly emerging technology which holds promise for diagnosis of disease and discovery of mechanisms linked to disease processes [13, 14], metabolomics approaches based on serum or urine samples have shown potential of establishing useful research tools for male infertility [15, 16]. Even though the metabolomics changes in seminal plasma, which is the most valuable sample type that mediates the chemical surrounding of sperm and the chemical function of the ejaculate, are still unclear. Seminal plasma is also a favorable noninvasive material which can provide the direct understanding of the dysregulated metabolism in reproductive organs, and may have great potential in the diagnosis of UMI.

In this study, we used a gas chromatography-mass spectrometry (GC-MS) based metabolomics approach to obtain the metabolic profiling, identified 153 seminal plasmatic metabolites from UMI patients. A robust OPLS-DA model based on these identified metabolites was able to distinguish 82% of the UMI patients from healthy controls with a specificity of 92%. In this model, 44 metabolites were found differentially expressed in UMI subjects. Our finding provides new perspective for diagnosis of UMI.

## Materials and methods

### Study population

The study was conducted in full compliance with government policies and the Helsinki Declaration, and the procedures were approved by institutional ethics committee of Nanjing Medical University. The subjects were the volunteers sequentially recruited from affiliated hospitals of Nanjing Medical University (NJMU). Every participant was fully informed about the purpose of this research, and written consent was obtained from each participant. All participants were ethnically Han Chinese. A questionnaire was used to collect information including personal background, lifestyle factors, occupational and environmental exposures, genetic risk factors, sexual and reproduction status, medical history, and physical activity. Cases included in this study were UMI with normal semen quality in accordance with WHO guidelines and normal physical as well as endocrine status. They were husbands of couples who attended the affiliated hospitals of NJMU because of conception failure for at least 12 months. Their wives had undergone a series of clinical tests and their reproductive function was evaluated as normal. The men had undergone complete historical and physical examinations and measurement of hormones as well as routine semen analysis; their partners had undergone complete historical and physical examinations, including conventional gynecological examination, serum hormone level measurements, tubal assessment, ovarian function testing, hysteroscopy, β-ultrasonic examination, immunologic test, microbial inspection, and karyotype analysis. Fertile controls (FC) in the present study were fertile men recruited and sampled at the same period as the UMI cases. They were from the early pregnancy registry of the same hospitals who were in the third month following a successful pregnancy. They were healthy men with normal reproductive function, prospectively confirmed by them fathering healthy babies 6~8 months later. Eighty UMI cases and 80 matched FCs were randomly included in training set; 50 UMI cases and 50 matched FC were randomly included in the validation set.

### Semen collection, analysis and storage

Semen samples were obtained in private by masturbation into a sterile wide-mouth and metal-free glass container after an at least 3-day abstinence. Routine semen analyses were performed using a computer aided semen analyzer (CASA) (WLJY 9000, Weili New Century Science and Tech Dev., China), while referring to guidelines in the fourth edition World Health Organization (WHO) Laboratory Manual for the Examination of Human Semen. After collection, the semen was liquefied at 37°C for 30 min and then analyzed in accordance with WHO guidelines; variables examined included semen volume, concentration, number per ejaculum, motility, vitality, progression, and motion parameters. Strict quality control measures were enforced throughout the entire study. Semen samples were assessed twice, and the counting procedure was automatic and performed by two technicians according to WHO recommendations. CASA results were compared directly with manual assessments using an improved Neubauer hemocytometer over a wide range of sperm concentrations. For sperm motility analysis, we used prepared videotapes to ensure that all motile spermatozoa were being identified using the current and consistent setup parameters.

After CASA analysis, each semen sample was prepared by Percoll gradient methods for separating viable spermatozoa and seminal plasma in the laboratory. Then all samples were stored at -80°C prior to GC-MS metabolic analysis.

### Sample preparation and metabolic profiling

The metabolic analysis and data processing were performed basically according to previous report [17]. Briefly, a 25 μl mixture of chloroform, methanol, and water (2:5:2) was added into 25 μl of seminal plasma and the samples were vortexed for 1 min. The samples were placed at -20°C for 20 min to extract metabolites, followed by centrifugation at 12,000 rpm for 10 min, and the liquid layer was transferred into a new tube. The residue was extracted with 50 μl of methanol using the homogenizer for 10 min followed by centrifugation at 12,000 rpm for 10 min. The supernatant was then combined with the previous extraction. After vortexing, a volume of 100 μl mixture was transferred to a glass vial spiked with internal standards (10 μl heptadecanoic acid at 1 mg/ml and 4-chlorophenylalanine at 0.3 mg/ml). Then the samples were vacuum dried at room temperature. The residue was chemically derivatized with a two-step procedure and then analyzed with Pegasus HT system (Leco Corporation, St Joseph, USA) coupled with an Agilent 6890N gas chromatography. Briefly, a 1 μl derivate was injected in splitless mode at 270°C. Helium was used as carrier gas; the flow rate was 1.0 ml/min. The transfer interface and ion source were set to 270°C and 220°C respectively. The oven temperature was set as a program which started at 80°C for 2 min, and then ramped to 180°C at a rate of 10°C/min, to 230°C with a rate of 6°C/min, finally to 295°C with a rate of 40°C/min and held for 8 min. MS data were acquired with m/z range of 30–600 at an acquisition rate of 20 spectrum/second. Compound identification for GC-TOFMS was performed by comparing the mass fragments with NIST Standard mass spectral databases using ChromaTOF software with a similarity of more than 70% and verified by available reference compounds.

### Quality control

An aliquot of 5 μl of each seminal plasma sample was pooled as the quality control sample. The QC samples were stored at -80°C and prepared once for each batch, and were injected every 10 samples in the GC-MS sequence. Responses of internal standards were used for creating a QC chart, those samples where deviation of internal standards were over control limit of 3 times of relative standard deviation (RSD) were re-analyzed. RSD of compounds in each QC sample were calculated; compounds that possessed an RSD over 30% were obsoleted.

### Statistical analysis

Fisher's exact test was used to analyze the differences of categorical variables such as drinking and smoking status between cases and controls. T-test was used to analyze for differences in continuous variables such as age and body mass index (BMI). Student-T tests were engaged to identify the differential seminal plasma metabolites between cases and controls, the data were log-transformed when appropriate. To obtain statistically robust results, the Bonferroni method was employed for correcting for multiple comparisons.

Those statistical methods above mentioned were all carried out within R programming language. Principal component analysis (PCA) and orthogonal partial least squares discriminant analysis (OPLS-DA) models were performed with SIMCA-P+ 13.5 (Umetrics, Umea, Sweden).

## Results

### Characteristics of the study population

As shown in Table 1, cases and controls were well matched for age, BMI, drinking status, and smoking status. No significant difference was observed between the two groups (Table 1).

**Table 1:** Summary description of the subjects used in this study.

Variables	Training Set (n = 160)		Validation Set (n = 100)		Total (n = 260)	
	Case (n = 80)	Control (n = 80)	Case (n = 50)	Control (n = 50)	Case (n = 130)	Control (n = 130)
**Age (years, mean ± s.d.)**	29.47 ± 3.78	29.32 ± 3.87	29.77 ± 4.79	30.80 ± 4.46	29.59 ± 4.18	29.89 ± 4.15
**BMI (mean ± s.d.)**	23.64 ± 2.66	23.07 ± 2.83	23.25 ± 2.87	23.09 ± 3.05	23.49 ± 2.74	23.08 ± 2.91
**Smoking [n (%)]**						
** Ever**	33 (41.25%)	36 (45.00%)	26 (52.00%)	30 (60.00%)	59 (45.40%)	66 (50.77%)
** Never**	47 (58.75%)	44 (55.00%)	24 (48.00%)	20 (40.00%)	81 (62.31%)	64 (49.23%)
**Drinking [n (%)]**						
** Yes**	39 (48.75%)	34 (42.50%)	22 (44.00%)	29 (58.00%)	61 (46.92%)	63 (48.8%)
** No**	41 (51.25%)	46 (57.50%)	28 (56.00%)	21 (42.00%)	69 (53.08%)	67 (51.3%)

### Seminal plastic metabolomics profile

**Fig 1:**
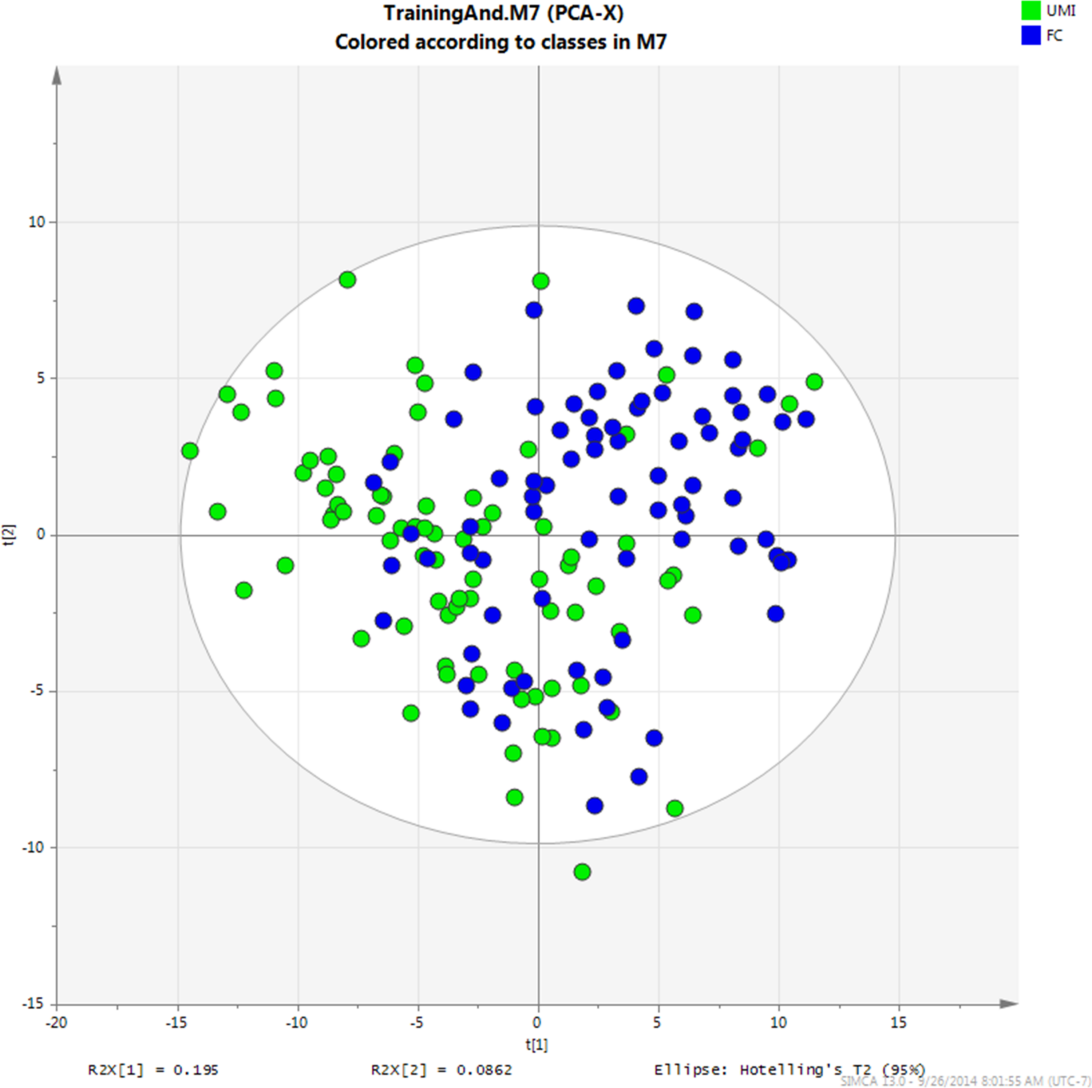
PCA score plot for training set.

A total of 333 metabolites were obtained from metabolic profiling of seminal plasma. 153 metabolites were identified by library matching, and 96 of those were validated by local standards. After application of QC criteria, 136 metabolites were found with RSD < 0.3. Ninety compounds of those were identified, and 70 were confirmed by local standards. A PCA model was created with a learning set using the 136 metabolites. PCA score plots (Fig 1) showed the separation trend between UMI and FS groups.

### Comparison of OPLS-DA models created with metabolomics data and seminal quality parameter data

**Fig 2:**
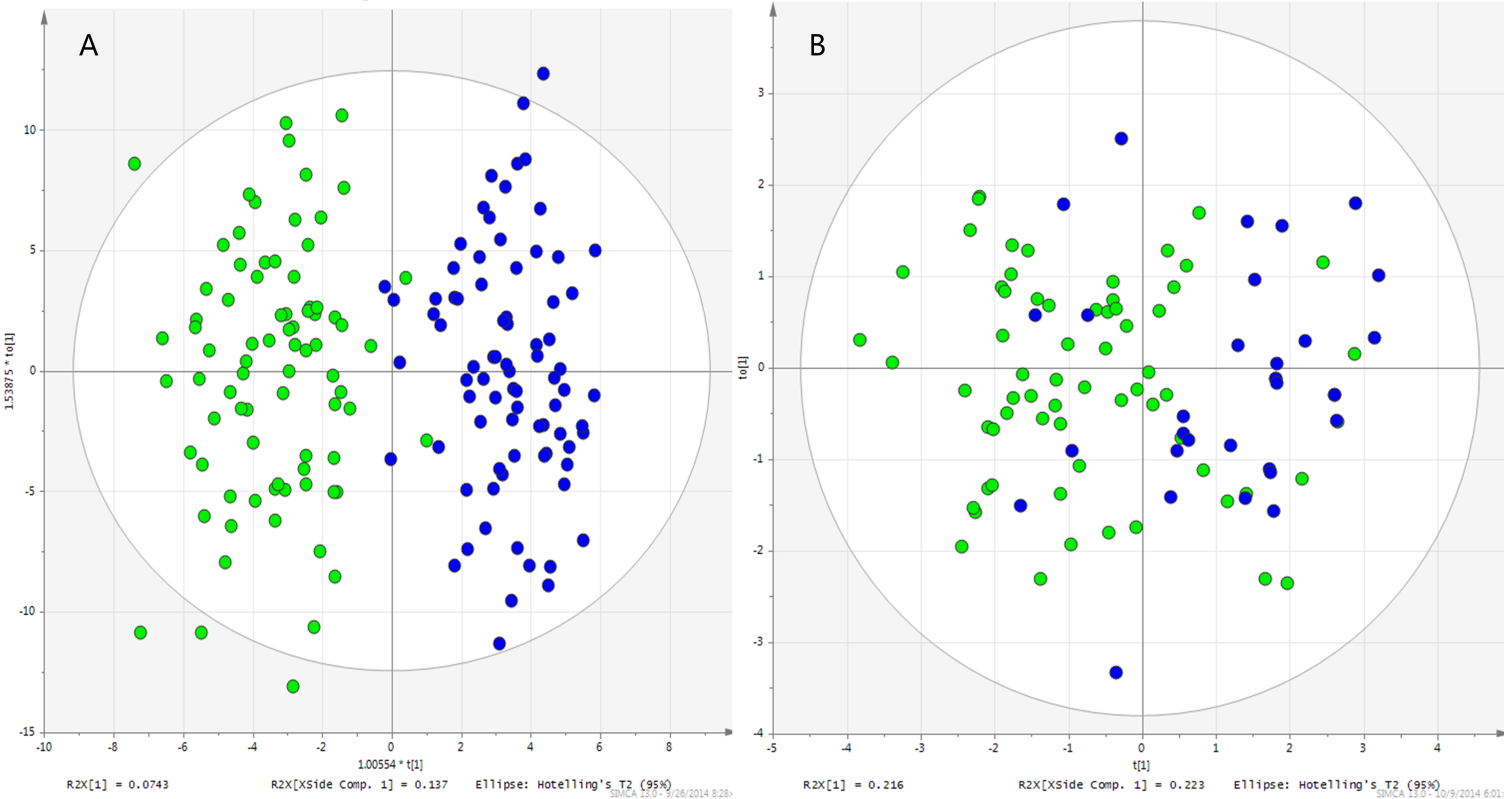
OPLS-DA score plot for training set. A. Generated with metabolomic profiling data; B. Generated with quality parameter of seminal plasma data.

Subsequently, OPLS-DA models were established and demonstrated satisfactory modeling and predictive abilities with 1 predictive component and 2 orthogonal components (R^2^Xcum = 0.333, R^2^Ycum = 0.833, Q2cum = 0.634) with metabolic profiling data (Fig 2A). As a comparison, quality parameters of seminal plasma, including semen volume, concentration, number per ejaculum, motility, vitality, progression, and motion parameters, were used for constructing another OPLS-DA model (R^2^Xcum = 0.360, R^2^Ycum = 0.423, Q2cum = 0.331). The score plot showed a poorer discriminate efficiency (a sensitivity of 82% and specificity of 92%) than that created with metabolomics data (a sensitivity of 98% and specificity of 95%) (Fig 2B).

### OPLS-DA model was validated by an independent sample set

**Fig 3:**
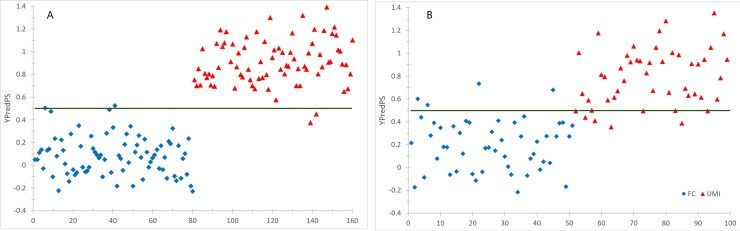
Classification result by OPLS-DA model. A. training set; B. validation set.

Furthermore, an independent set of samples were profiled as the validation set which consisted of 50 UMI and 50 FC. Profiling data from the validation set were used to test the prediction ability of the established OPLS-DA model. In the Y prediction scores plot, 41 out of 50 UMI samples and 46 out of 50 control samples in validation groups were correctly assigned to proper class, using a cutoff value of 0.5 (Fig 3, the Y value was set at 1 for UMI, and 0 for controls in the learning group). This result showed the ability of the OPLA model to predict the unknown samples to the right groups with a sensitivity of 82% and specificity of 92%. As all these UMI were diagnosed as fertile male under current WHO seminal plasmatic parameter standards, the OPLS-DA model is of great significance in the diagnosis of UMI.

### Seminal plastic biomarkers of UMI

Significantly altered metabolites in seminal plasma with a VIP threshold (VIP > 1) in above mentioned OPLS-DA model, as well as the Mann-Whitney U test between UMI and FC, were selected and summarized in Table 2. Among these 44 differential metabolites, 37 were confirmed by reference standards as shown in Table 2. Variations of these differential metabolites were described by fold changes where UMI subjected to FC. *P*-values and fold changes in validation group were also listed, and coincided well with those in the training set.

**Table 2:** Differential metabolites between UMI and FC.

Metabolites	VIP value	Training set		Validation set	
		*P*	Fold change ^c^	*P*	Fold change ^c^
**Itaconic acid** ^**a**^	2.14	7.1E-13	0.68	9.1E-06	0.73
**Urea** ^**a**^	1.97	6.0E-11	14.95	1.6E-07	4.97
**4-Hydroxyphenylacetic acid** ^**a**^	1.87	6.6E-10 ^b^	0.19	8.9E-10 ^b^	0.2
**Glutamic acid** ^**a**^	1.8	3.8E-09	0.54	6.8E-07	0.62
**Threonic acid** ^**a**^	1.79	9.1E-10 ^b^	0.74	2.2E-06 ^b^	0.73
**Glucose** ^**a**^	1.77	6.5E-09	12.46	2.3E-07	13.97
**Pyroglutamic acid** ^**a**^	1.77	6.9E-09	0.68	2.0E-06	0.73
**Valine** ^**a**^	1.77	7.1E-09	0.73	2.8E-07	0.76
**Isoleucine** ^**a**^	1.74	1.3E-08	0.73	9.6E-07	0.76
**Mannose** ^**a**^	1.71	2.6E-08	2.42	1.5E-07	4.25
**Glyceric acid** ^**a**^	1.71	3.9E-11 ^b^	0.44	1.1E-06 ^b^	0.59
**Hydroxyacetic acid** ^**a**^	1.7	3.1E-08	0.61	5.4E-05	0.62
**Threonine** ^**a**^	1.67	5.6E-08	0.74	2.2E-06	0.77
**Serine** ^**a**^	1.63	1.3E-07	0.77	1.5E-06	0.8
**Gluconic acid** ^**a**^	1.62	1.6E-07	2.07	3.3E-06	7.85
**Galactose** ^**a**^	1.61	2.0E-07	0.71	2.5E-03	0.76
**Alanine** ^**a**^	1.6	2.1E-07	0.63	9.6E-05	0.75
**Spermidine** ^**a**^	1.59	2.9E-07	1.51	2.8E-03	1.64
**2,3-Dihydroxy-Propanoic acid**	1.59	2.9E-07	0.79	3.4E-03	0.84
**Uridine** ^**a**^	1.52	3.2E-08 ^b^	0.61	4.2E-04 ^b^	0.65
**Ethanolamine** ^**a**^	1.48	1.8E-06	0.78	5.4E-03	0.81
**Glutamine** ^**a**^	1.48	1.9E-06	5.18	1.6E-06	2.55
**Aspartic acid** ^**a**^	1.48	2.0E-06	0.33	7.6E-05	0.51
**Phenylalanine** ^**a**^	1.47	2.2E-06	0.76	2.8E-03	0.8
**Proline** ^**a**^	1.46	2.7E-06	0.46	8.1E-05	0.6
**Inositol** ^**a**^	1.42	5.2E-06	0.78	9.8E-04	0.81
**Cadaverine** ^**a**^	1.42	5.6E-06	0.37	5.0E-05	0.43
**Aminomalonic acid**	1.41	5.8E-06	0.77	1.1E-02	0.76
**Tryptophan** ^**a**^	1.39	8.6E-06	0.68	6.1E-03	0.69
**Rhamnose** ^**a**^	1.38	1.1E-05	0.76	6.5E-04	0.75
**Xanthine** ^**a**^	1.37	1.2E-05	0.88	1.6E-01	0.88
**N-Acetyl glucosamine** ^**a**^	1.35	1.6E-05	0.81	6.1E-03	0.79
**Asparagine** ^**a**^	1.35	3.4E-06 ^b^	0.77	7.5E-04 ^b^	0.76
**Dopamine**	1.27	5.2E-05	0.92	1.6E-04	0.87
**Glycine** ^**a**^	1.27	5.2E-05	0.8	5.2E-04	0.76
**Hypoxanthine** ^**a**^	1.24	5.3E-06 ^b^	0.54	1.9E-03 ^b^	0.63
**Spermine** ^**a**^	1.21	2.1E-05 ^b^	4.48	8.5E-03 ^b^	2.51
**Citric acid** ^**a**^	1.21	4.8E-06 ^b^	0.8	6.4E-05 ^b^	0.78
**Xylitol** ^**a**^	1.2	1.4E-05 ^b^	0.79	2.8E-03 ^b^	0.75
**Dehydroascorbic acid**	1.16	2.3E-04	4.49	1.5E-02	3.59
**3-Hydroxyisobutyric acid**	1.14	3.1E-04	0.95	9.0E-01	1.02
**Glycerol 2-phosphate**	1.09	5.4E-04	0.72	1.2E-04	0.76
**Threitol** ^**a**^	1.08	1.6E-07 ^b^	0.59	5.1E-07 ^b^	0.68
**Dithiothreitol** ^**a**^	1.06	7.7E-04	0.9	1.8E-01	0.67

^a^ Metabolites confirmed by local standards.

^b^ Data was log transformed before U test.

^c^ Folder changes of metabolites were calculated with mean value in FC and UMI group, while those with log transformed were calculated with the median. A fold change more than 1 means an increase in UMI group, whereas less than 1 represents a decrease.

### Correlations between seminal plasmatic metabolites and quality parameters of seminal plasma

Pearson correlation coefficient was calculated with metabolic profiling data and quality parameters of seminal plasmatic, including semen volume, concentration, number per ejaculum, motility, vitality, progression, and motion parameters. Significant correlated metabolites and quality parameters were listed in Table 3.

**Table 3:** Correlations between seminal plastic metabolites and semen parameters.

	AT		pH		SPMS		VCL		VSL		VAP		BCF		SC	
	*P*	r	*P*	r	*P*	r	*P*	r	*P*	r	*P*	r	*P*	r	*P*	r
4-Hydroxyphenylacetic acid	6E-01	0.05	2E-06	-0.42	8E-03	0.25	6E-03	-0.25	1E-02	-0.23	8E-03	-0.24	1E-01	0.15	3E-20	0.72
Fumaric acid	4E-06	0.41	1E-03	-0.29	2E-04	0.34	4E-05	-0.37	1E-05	-0.39	1E-05	-0.40	3E-05	0.38	4E-14	0.63
Mannose	3E-04	-0.33	5E-01	0.06	4E-04	-0.32	3E-04	0.33	8E-05	0.36	2E-04	0.33	8E-08	-0.47	1E-10	-0.55
Glycerol phosphate	8E-01	-0.03	5E-01	-0.06	5E-01	0.06	1E-01	-0.14	2E-01	-0.12	2E-01	-0.13	5E-01	0.06	2E-08	0.49
Itaconic acid	3E-02	0.20	6E-04	-0.31	7E-04	0.31	5E-05	-0.36	2E-04	-0.33	8E-05	-0.36	2E-02	0.22	3E-08	0.49
Glucose	6E-05	-0.36	6E-01	0.05	7E-06	-0.40	7E-05	0.36	4E-06	0.41	1E-05	0.39	9E-09	-0.50	5E-08	-0.48
Glutamic acid	1E-06	0.43	1E-05	-0.39	3E-08	0.49	3E-08	-0.48	4E-07	-0.45	1E-07	-0.46	9E-08	0.47	8E-08	0.47
3-Phenyllactic acid	9E-06	0.40	3E-03	-0.28	7E-08	0.47	8E-08	-0.47	1E-07	-0.47	5E-08	-0.48	5E-07	0.44	1E-07	0.47
Lactic acid	4E-02	0.19	6E-01	-0.05	8E-03	0.24	3E-01	-0.10	4E-02	-0.19	1E-01	-0.15	1E-02	0.22	1E-07	0.47
Aspartic acid	2E-06	0.42	5E-05	-0.37	4E-09	0.51	3E-08	-0.49	9E-08	-0.47	3E-08	-0.49	1E-08	0.50	4E-07	0.45
Glycerol 2-phosphate	1E-01	0.15	2E-01	-0.11	2E-04	0.34	2E-05	-0.39	4E-06	-0.41	1E-06	-0.43	2E-02	0.21	2E-06	0.42
Ethanolamine	1E-01	0.14	4E-01	-0.08	2E-02	0.21	7E-04	-0.31	4E-03	-0.26	2E-03	-0.29	1E-01	0.14	3E-06	0.42
Cadaverine	9E-04	0.30	3E-02	-0.20	3E-06	0.42	1E-05	-0.39	2E-05	-0.39	6E-06	-0.41	9E-04	0.30	4E-06	0.41
Pyroglutamic acid	7E-04	0.31	2E-04	-0.34	3E-07	0.45	2E-08	-0.49	5E-07	-0.45	6E-08	-0.48	4E-05	0.37	2E-05	0.38
Valine	3E-04	0.33	4E-04	-0.32	5E-08	0.48	8E-09	-0.50	9E-08	-0.47	2E-08	-0.49	2E-05	0.38	2E-05	0.38
Isoleucine	2E-04	0.34	1E-03	-0.30	7E-08	0.47	2E-08	-0.49	2E-07	-0.46	3E-08	-0.48	3E-05	0.38	6E-05	0.36
Alanine	4E-05	0.37	2E-04	-0.34	4E-09	0.51	2E-08	-0.49	2E-08	-0.49	5E-09	-0.51	9E-07	0.44	7E-05	0.36
Threonine	3E-04	0.33	4E-04	-0.32	9E-08	0.47	9E-09	-0.50	8E-08	-0.47	1E-08	-0.49	1E-05	0.39	1E-04	0.35
Proline	6E-07	0.44	1E-04	-0.35	3E-09	0.52	3E-08	-0.48	7E-08	-0.47	2E-08	-0.49	1E-07	0.47	1E-04	0.35
Urea	1E-05	-0.39	6E-02	0.18	3E-05	-0.38	4E-04	0.32	1E-03	0.30	9E-04	0.30	1E-03	-0.29	1E-04	-0.35
Tryptophan	2E-06	0.43	2E-03	-0.29	9E-09	0.50	3E-08	-0.48	4E-08	-0.48	2E-08	-0.49	5E-08	0.48	3E-04	0.33
Threitol	2E-03	0.29	2E-03	-0.28	4E-06	0.41	4E-05	-0.37	2E-04	-0.34	2E-04	-0.34	9E-04	0.30	4E-04	0.32
Glycine	1E-05	0.40	1E-02	-0.23	4E-07	0.45	2E-08	-0.49	3E-08	-0.49	8E-09	-0.50	1E-06	0.43	5E-04	0.32
Serine	1E-03	0.30	4E-03	-0.27	1E-06	0.43	3E-08	-0.49	3E-07	-0.45	5E-08	-0.48	2E-04	0.34	9E-04	0.30
Aminomalonic acid	7E-07	0.44	1E-01	-0.15	5E-06	0.41	2E-04	-0.33	4E-06	-0.41	6E-06	-0.40	1E-05	0.39	3E-03	0.27
Phenylalanine	2E-04	0.34	1E-02	-0.24	8E-07	0.44	1E-06	-0.43	3E-06	-0.42	8E-07	-0.44	9E-05	0.35	4E-03	0.27
Homocysteine	8E-06	0.40	2E-02	-0.22	2E-09	0.52	3E-07	-0.45	7E-08	-0.47	4E-08	-0.48	1E-08	0.50	6E-03	0.25
Ribonic acid	2E-04	0.34	5E-02	-0.18	8E-08	0.47	3E-04	-0.33	2E-04	-0.34	2E-04	-0.34	2E-05	0.39	7E-03	0.25
Arabionse	2E-04	-0.34	3E-01	0.09	3E-07	-0.45	3E-04	0.33	4E-07	0.45	5E-07	0.44	7E-06	-0.40	9E-03	-0.24
Maltose	3E-04	-0.33	8E-02	0.16	2E-05	-0.38	3E-05	0.37	3E-05	0.38	1E-05	0.39	2E-05	-0.39	1E-02	-0.23
Lysine	2E-04	0.33	2E-02	-0.21	4E-06	0.41	4E-07	-0.45	6E-07	-0.44	2E-07	-0.46	7E-06	0.40	6E-02	0.18
3-Hydroxyisobutyric acid	8E-05	0.36	1E-01	-0.14	4E-07	0.45	3E-02	-0.20	1E-04	-0.34	2E-04	-0.34	2E-03	0.28	7E-02	0.17

AT: abstinence time; SPMS: slowly progressive motile sperm; VCL: eurvilinear velocity; VSL: straight-line velocity; VAP: average path velocity; BCF: beat-cross frequency; SC: sperm concentration.

### Metabolic pathway changes in UMI

Metabolic pathway analysis were carried out with MetaboAnalyst [18]. The seminal plastic biomarkers of UMI were input as data source. Seven from 80 homo-sapiens (human) pathways were significantly changed as shown in Table 4.

**Table 4:** Result from pathway analysis.

Pathway	Total ^a^	Hits ^b^	Raw *p* ^c^	Holm p ^d^	Impact ^e^
**Aminoacyl-tRNA biosynthesis**	75	13	1.57E-10	1.26E-08	0.17
**Nitrogen metabolism**	39	7	3.52E-06	2.78E-04	0.01
**Glutathione metabolism**	38	6	4.10E-05	3.19E-03	0.08
**Arginine and proline metabolism**	77	8	4.46E-05	3.43E-03	0.21
**Alanine, aspartate and glutamate metabolism**	24	5	4.74E-05	3.60E-03	0.75
**Cyanoamino acid metabolism**	16	4	1.38E-04	1.03E-02	0.00
**Glycine, serine and threonine metabolism**	48	6	1.59E-04	1.18E-02	0.42

^a^ Total is the total number of compounds in the pathway.

^b^ Hits is the actually matched number from the user uploaded data.

^c^ Raw p is the original p value calculated from the enrichment analysis.

^d^ Holm p is the p value adjusted by Holm-Bonferroni method.

^e^ Impact is the pathway impact value calculated from pathway topology analysis.

## Discussion

UMI is considered a complicated disease with a genetic, proteomic, and metabolic basis. Several causes of UMI are still unknown. Previous studies have shown that metabolic fingerprinting can be applied as a screening tool for male infertility problems. In our present study, using a GC-MS-based metabolite profiling platform, a robust OPLS-DA model was able to distinguish 82% of the UMI patients from FC, from which 44 metabolites were identified differentially expressed in UMI subjects. This finding was validated with the validation set, confirming the result was robust. Pearson correlation coefficient analysis found that the relationships between the concentrations of seminal plasmatic metabolites and seminal quality parameters, indicating the result was biologically relevant.

Metabolomics has been proven to be a promising tool in disease diagnosis. The applications of metabolic-based approaches in discriminate asthenozoospermia or oligoasthenozoospermia using seminal plasma and urine have been emerging recently, confirming metabolic approach has great potential in aiding in the routine diagnosis of male infertility. However, asthenozoospermia or oligoasthenozoospermia can be identified by rountine semen analysis, there is a great need to apply metabolic approach in the identification of biomarkers related to UMI and further diagnosis of this disease. A robust OPLS-DA model based on these identified metabolites was built which was able to distinguish 82% of the UMI patients from healthy controls, from which the concentration of 44 metabolites were found aberrantly expressed in UMI subjects. Our findings, which enable the diagnostic of UMI using merely seminal plasmatic specimen, indicating our finding has great clinical significance, and will be a possible tool for identify UMI for couples that decide to conceive a baby.

**Fig 4:**
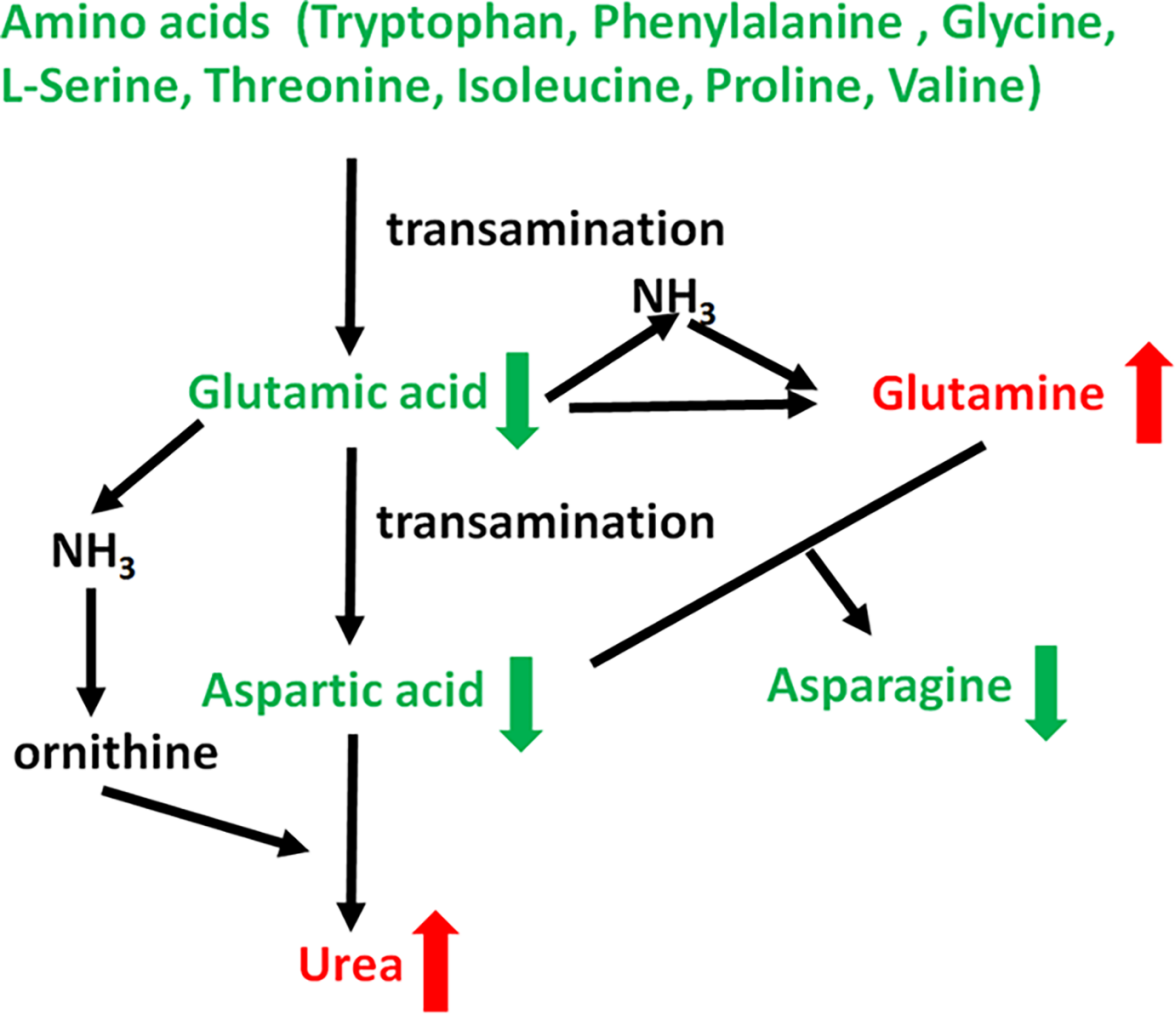
Sketch map of metabolic changes in UMI.

The biomarkers identified in metabolomics may be related to the pathogenesis of disease, and may provide novel therapeutic targets of disease [19, 20]. Autoimmune infertility and deficient sperm function such as reactive oxygen species and fertilization defects were considered to be the causes of UMI, but the metabolic causes of UMI are still unknown [3]. Fig 4 was built based on the major findings of pathway enrichment revealed in Table 3. A notable metabolic feature of UMI patients was the remarkably disturbed amino acid metabolism. Various amino acids, including tryptophan, phenylalanine, glycine, serine, threonine, isoleucine, proline, and valine significantly decreased in seminal plasma of UMI, indicating a possible increased consumption of these amino acids. Notably, they all can be converted to glutamic acid by transamination, which can further produce ammonia. In the UMI seminal plasma, we even found decreased glutamic acid, indicating the potential ammonia burden in UMI. The human body controls the ammonia concentration in an accepted level by consumption of ammonia to generate glutamine and urea. Urea is eliminated from body by urination, and glutamine is not toxic. Interestingly, among the numerous decreased metabolites in the enriched pathways related to amino acid metabolism in UMI, we found urea and glutamine increased significantly in UMI seminal plasma, indicating the major metabolic signature of UMI seminal plasma is the increased catabolism of various amino acids. Furthermore, these changed amino acids might play roles in male reproduction. Aminoacyl-tRNAs are substrates for translation and are pivotal in determining how the genetic code is interpreted as amino acids. Some of these amino acids such as tryptophan, serine, and glycine can form aminoacyl-tRNAs. We also found the concentrations of tryptophan, phenylalanine, glycine, serine, threonine, isoleucine, proline, and valine all showed positive relationships with sperm counts, while the concentration of their deamination product urea was negatively related to sperm counts.

In this study, 4-hydroxyphenylacetic acid was identified as a key metabolite in differentiating the UMI and controls, and its concentration in seminal plasma was positively related to sperm counts. Imbalance between the oxidant burden and antioxidant defense system has aroused great attention on UMI, because it exerts a critical pathological effect on sperm function through sperm DNA damage in the nucleus and mitochondria as well as by inducing lipid peroxidation in the sperm plasma membrane [21]. A natural antioxidant defense system consists of radical scavengers, chain breaking antioxidants, and ROS-metabolizing enzymes offering protection against ROS. Vitamin C, uric acid, tryptophan, and taurine are accepted radical scavengers [22]. The lack of radical scavengers in seminal plasma contributes to UMI. Notably, it is widely reported that 4-hydroxyphenylacetic acid and related phenolic compounds exert an anti-oxidant effect [23–25]. Therefore, one reasonable mechanism underlying the involvement of 4-hydroxyphenylacetic acid in UMI is the lack of this anti-oxidant chemical for radical clearage. 4-hydroxyphenylacetic acid is an oxidative deaminated metabolite of p-tyramine and also a metabolite of tyrosine via enteric bacteria. However, this metabolite is not only endogenous, it can also be supplemented directly from foods such as olive oil [22, 23]. Therefore, there may be a convenient therapeutic intervention for UMI by nutrition, which needs further investigation.

Strengths of the present study include its sample size and the two-stage population study design, as well as the use of well-validated metabolic approach which can provide reliable and accurate metabolic data. Future studies need to verify the metabolic changes in this study and their effects on male fertility both in *in vitro* and *in vivo* and to elucidate the underlying mechanism. The application of metabolomics in discriminating more clinical types of male reproductive abnormalities is also needed in the future.

In conclusion, using a GC-MS-based metabolite profiling platform, a robust OPLS-DA model was established for distinguishing UMI patients from FC. From the differentially expressed metabolites in UMI subjects, we identified the metabolic signature of UMI. Our findings provide new potential diagnosis biomarker and treatment target of UMI and may improve the current treatment and future clinical practice of assisted reproductive technology. With further study of linkage between metabolic pattern and other environmental risk factors, such as diet pattern, oxidative stress, etc., it may prompt new couples how to increase chances to have a baby.
